# Can inorganic phosphate explain sag during unfused tetanic contractions of skeletal muscle?

**DOI:** 10.14814/phy2.13043

**Published:** 2016-11-24

**Authors:** Ian C. Smith, Catherine Bellissimo, Walter Herzog, A. Russell Tupling

**Affiliations:** ^1^ Human Performance LabUniversity of CalgaryCalgaryAlbertaCanada; ^2^ Department of KinesiologyUniversity of WaterlooWaterlooOntarioCanada

**Keywords:** Crossbridge cycling, EDL, fast‐ and slow‐twitch muscle, muscle stimulation, simulated contraction, soleus, twitch kinetics

## Abstract

We test the hypothesis that cytosolic inorganic phosphate (P_i_) can account for the contraction‐induced reductions in twitch duration which impair summation and cause force to decline (sag) during unfused tetanic contractions of fast‐twitch muscle. A five‐state model of crossbridge cycling was used to simulate twitch and unfused tetanic contractions. As P_i_ concentration ([P_i_]) was increased from 0 to 30 mmol·L^−1^, twitch duration decreased, with progressive reductions in sensitivity to P_i_ as [P_i_] was increased. When unfused tetani were simulated with rising [P_i_], sag was most pronounced when initial [P_i_] was low, and when the magnitude of [P_i_] increase was large. Fast‐twitch extensor digitorum longus (EDL) muscles (sag‐prone, typically low basal [P_i_]) and slow‐twitch soleus muscles (sag‐resistant, typically high basal [P_i_]) were isolated from 14 female C57BL/6 mice. Muscles were sequentially incubated in solutions containing either glucose or pyruvate to create typical and low P_i_ environments, respectively. Twitch duration was greater (*P *<* *0.05) in pyruvate than glucose in both muscles. Stimuli applied at intervals approximately three times the time to peak twitch tension resulted in sag of 35.0 ± 3.7% in glucose and 50.5 ± 1.4% in pyruvate in the EDL (pyruvate > glucose; *P *<* *0.05), and 3.9 ± 0.3% in glucose and 37.8 ± 2.7% in pyruvate in the soleus (pyruvate > glucose; *P *<* *0.05). The influence of P_i_ on crossbridge cycling provides a tenable mechanism for sag. Moreover, the low basal [P_i_] in fast‐twitch relative to slow‐twitch muscle has promise as an explanation for the fiber‐type dependency of sag.

## Introduction

Cooper and Eccles (1930) first investigated how changing the time interval between stimuli influences the shape of tension records in cat hind limb muscles. They found that peak‐to‐peak tension initially rose during the first few impulses of unfused tetanic contractions of the gastrocnemius, extensor digitorum longus (EDL), and soleus muscles. However, tension declined later in the tetani of the gastrocnemius and EDL, but not in the soleus. The presence or absence of these declines in force, later termed “sag”, have been used to classify motor units as either fast or slow, respectively, with good agreement with other functional and histochemical means of differentiating fast and slow motor units (Burke et al. 1973).

Sag results from highly effective summation early in the unfused tetanus, which becomes less effective as the contraction duration decreases during the unfused tetanus. The reductions in contraction duration affect both the time to peak tension and the relaxation times. While much work has been done examining the nature of sag, little is known about the underlying cellular mechanism. Electromyography signals measured before and after the induction of sag ruled out failure of neuromuscular transmission as a possible mechanism (Burke et al. 1976). As fast and slow motor units within a single muscle can differ in their exhibition of sag, connective tissue properties have also been discounted as a possible mechanism (Burke 1990). It has been concluded that sag is an inherent property of the muscle fibers (Burke 1990). The causative factor of sag does not require an active population of crossbridges, as depressed force levels remain for several hundred milliseconds if an unfused tetanus is interrupted following the development of sag (Burke 1990). Similarly, if unfused tetani are applied in rapid succession, all contractions will achieve a similar tension plateau, but the high initial peak is attenuated or absent in all contractions subsequent to the first (Celichowski et al. 2005).

The progressive reductions in contraction duration that lead up to and persist with sag have been proposed to result from reductions in the duration of the force‐producing crossbridge states relative to the duration of nonforce‐producing crossbridge states (Cooper and Eccles 1930; Burke et al. 1973, 1976; Carp et al. 1999; Raikova et al. 2008). Reductions in contraction duration are also seen with repeated twitch contractions (Krarup 1981; Smith et al. 2014). Presently, acceleration in the rate of Ca^2+^ uptake into the sarcoplasmic reticulum is the favored mechanism to explain the reductions in contraction duration (Burke 1990; Carp et al. 1999; Brown and Loeb 2000; Celichowski et al. 2005, 2011; Krutki et al. 2006; Tupling 2009; Tsianos and Loeb 2013). Consistent with this attribution, Brown and Loeb (2000) used the assumption that Ca^2+^ removal is accelerated during contraction to model activation dynamics for a fast‐twitch muscle, and accurately predicted the force profiles seen during actual experiments. However, their model equated a reduction in the stimulation frequency during the contraction to an acceleration of cytosolic Ca^2+^ removal; an approach which cannot distinguish between crossbridge‐based and Ca^2+^‐based mechanisms of reducing contraction duration. This distinction is important because although removal of Ca^2+^ from the cytosol is necessary for relaxation, the rate of Ca^2+^ removal does not always determine the rate of relaxation in intact muscle (Johnson et al. 1997; Janssen et al. 2002; Little et al. 2012). Also problematic for a Ca^2+^‐based explanation for sag is the lack of empirical evidence demonstrating that the rate of Ca^2+^ uptake can be rapidly increased with brief contractile activity. Discordant with the Ca^2+^‐based theory of sag, we have recently demonstrated that contraction duration is reduced during repeated twitch contractions (Smith et al. 2014) and following brief tetanic contractions (Smith et al. 2013b) without an associated reduction in the duration of the intracellular Ca^2+^ transients in fast‐twitch mouse muscle.

Sag must be attributable to an aspect of excitation–contraction coupling which is downstream of the intracellular Ca^2+^ transient. This mechanism must possess the ability to rapidly increase the rate of crossbridge detachment during contractions, and correlate to the oxidative potential, fatigability, and various other fiber‐type properties to account for the fiber‐type dependence of sag (Burke et al. 1973). Inorganic phosphate (P_i_) accumulation fits both these criteria. Studies using both fast‐ and slow‐skinned fiber preparations have shown that increased P_i_ concentrations ([P_i_]) decrease the number of force‐generating crossbridges via a P_i_‐mediated reversal of the strong binding step (Hibberd et al. 1985; Palmer and Kentish 1994; Zhao and Kawai 1994; Coupland et al. 2001; Debold et al. 2004, 2006; Caremani et al. 2008), perhaps with an additional influence from a branch in the crossbridge cycle in which P_i_ facilitates the detachment of ADP‐bound, strained crossbridges (Kerrick and Xu 2004; Linari et al. 2010), and enhances relaxation rates (Tesi et al. 2002). Reducing the [P_i_] has been demonstrated to slow relaxation rates in both skinned fibers (Luo et al. 2002) and intact muscle (Phillips et al. 1993). Using a 3‐bead laser trap assay, Debold et al. (2013) demonstrated that the duration of crossbridge‐binding events is reduced by over 65% in the presence of 30 mmol·L^−1^ P_i_ compared to a P_i_‐free condition. The accumulation of P_i_ during the course of a contraction (Kushmerick and Meyer 1985; Challiss et al. 1989) should therefore be sufficient to result in sag. However, the effects of P_i_ on crossbridge function are comparable between fiber types (Tesi et al. 2002; Kerrick and Xu 2004), indicating that sag should be inducible in both fast‐ and slow‐twitch muscles. Why sag should appear in fast but not slow muscle may be explained by the fiber‐type‐dependent differences in [P_i_] at the onset of contraction, where P_i_ is markedly lower in fast muscles (0 to 3 mmol·L^−1^) than in slow muscles (4 to 7 mmol·L^−1^) at rest (Kushmerick et al. 1992; Phillips et al. 1993; Dahlstedt et al. 2000), and the sensitivity of muscle tension to changes in [P_i_] are greatest at [P_i_] near 1 mmol·L^−1^ (Cooke and Pate 1985; Pate et al. 1998).

Phillips et al. (1993) validated a method of reducing the basal cytosolic [P_i_] ([P_i_]_c_) in isolated intact muscle preparations by incubation in physiological salt solutions containing pyruvate rather than glucose which is typically used as an exogenous energy source. How changing the substrate might influence P_i_ levels has been discussed elsewhere (Daut and Elzinga 1988; Phillips et al. 1993). Briefly, P_i_ levels reflect the balance between ATP utilization and ATP production through oxidative phosphorylation and glycolysis. Supplying exogenous pyruvate is hypothesized to bypass the rate‐limiting step in glycolysis (i.e., phosphofructokinase) which is sensitive to [P_i_] (Passoneau and Lowry 1964). The higher pyruvate concentration increases mitochondrial redox potential, which in turn increases the cytosolic phosphorylation potential, and [P_i_]_c_ is thereby reduced.

**Figure 1:**
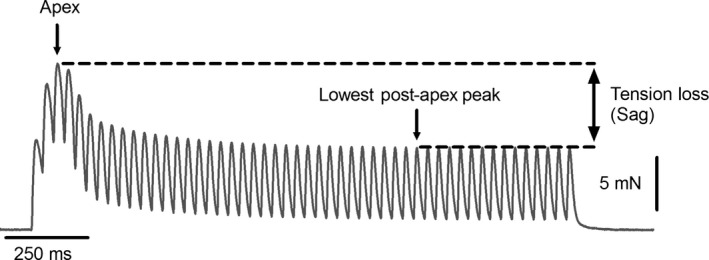
Sag was quantified as the tension lost between the apex and the lowest local peak that followed the apex.

The aim of this study was to determine if changes in cytosolic P_i_ could explain the changes in contraction duration that cause sag during unfused tetani. In this study, we present results from a five‐state model describing the effects of P_i_ on crossbridge cycling to demonstrate how P_i_ might influence crossbridge cycling to cause sag during unfused contractions. A depiction of sag and how it was quantified in this study is seen in Figure 1. We have also incubated intact mouse soleus and extensor digitorum longus muscles in pyruvate and glucose solutions to modify basal [P_i_], which induced changes in twitch kinetics and sag parameters consistent with a P_i_‐based mechanism for sag.

## Materials and Methods

### Modeled contractions

**Figure 2:**
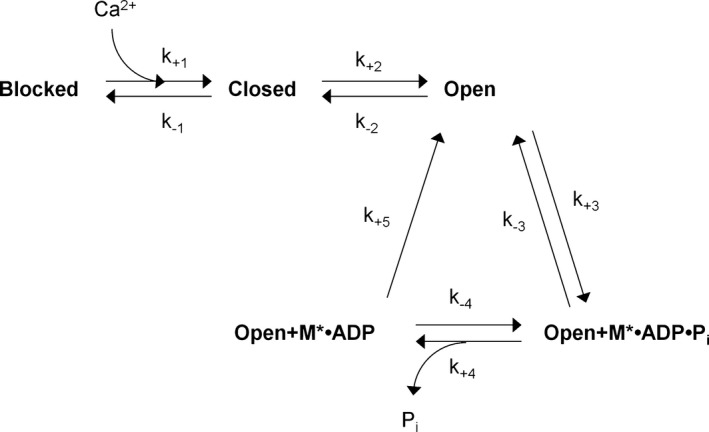
The chemo‐mechanical cycle used in the simulations.

The effects of intracellular P_i_ on twitch and unfused tetanic contractions were simulated by combining aspects of several existing models (Robertson et al. 1981; McKillop and Geeves 1993; Coupland et al. 2001). The resultant model incorporates the Ca^2+^‐dependent gating of myosin binding to actin, and inhibitory effects of phosphate on the maintenance of crossbridge binding. This model is depicted in Figure 2, and the rate constants used are shown in Table 1. Our model has five states – (1) the Blocked state, (2) the Closed state, (3) the Open state, (4) the Open·Myosin·ADP·P_i_‐bound state, and (5) the Open·Myosin·ADP‐bound state. States 1, 2, and 3 represent the different levels of thin filament activation which gate myosin attachment, while states 4 and 5 are considered to be force‐producing. Transitions between the Blocked and Closed states were determined by a Ca^2+^‐dependent rate constant of 1.15 × 10^8^M^−1·^s^−1^ (Robertson et al. 1981) for the forward step (k_+1_), and a rate constant of 227 s^−1^ (McKillop and Geeves 1993) for the reverse step (k_−1_). This resulted in an equilibrium constant for K_1_ of 16.0 at pCa 4.5, and 5.1 × 10^−4^ at pCa 9.0. The transitions between Closed and Open states were in equilibrium with K_2_ = 0.25 (McKillop and Geeves 1993). In contrast to the model of McKillop and Geeves, we elected to keep K_2_ constant regardless of Ca^2+^ availability and did not incorporate any alternative mechanism to account for the cooperative aspects of Ca^2+^ and crossbridge binding. This choice increased the sensitivity of our model to low Ca^2+^ concentrations. To compensate, resting Ca^2+^ was set to pCa 10 in our simulations, and the simulated Ca^2+^‐transients were adjusted to span a larger range of pCa values (see below). The rate of Myosin·ADP·P_i_‐binding to the Open state (k_+3_) was designated 1050 s^−1^ as this reproduced the P_i_‐dependency of Ca^2+^‐saturated, steady‐state forces seen at 30°C in Coupland et al. (2001), and the reverse rate (k_−3_) was 135 s^−1^ Coupland et al. (2001). The forward (k_+4_) and reverse (k_−4_) rates of P_i_ dissociation were the same as those used by Coupland et al. (2001). The transition from the Myosin·ADP state to the Open state (k_+5_) incorporated all the steps necessary for the myosin head to detach and re‐prime, it was considered irreversible, and it was rate‐limiting at 10 s^−1^ (Coupland et al. 2001). Our model did not account for changes in sarcomere length which occur in intact preparations, leading to the absence of a “shoulder” region during relaxation (see Huxley and Simmons 1970) in our modeled contractions. Thus, the relaxation properties of our modeled contraction better reflect the early linear phase of relaxation than the later “exponential” phase of relaxation. The system of differential equations describing the kinetic cycle of our model is shown below.

$$\text{dBlocked}\left(t\right)/dt=k_{-1}\times \text{Closed}\left(t\right)-k_{+1}\times \left[{\text{Ca}}^{2+}\right]\left(t\right)\times \text{Blocked}\left(t\right)$$

$$\text{dClosed}\left(t\right)/dt=k_{+1}\times \left[{\text{Ca}}^{2+}\right]\times \text{Blocked}\left(t\right)+k_{-2}\times \text{Open}\left(t\right)-\left(k_{-1}+k_{+2}\right)\times \text{Closed}\left(t\right)$$

$$\text{dOpen}\left(t\right)/dt=k_{+2}\times \text{Closed}\left(t\right)+k_{-3}\times \text{Open}\cdot M\cdot \text{ADP}\cdot P_{i}\left(t\right)+k_{+5}\times \text{Open}\cdot M\cdot \text{ADP}\left(t\right)-\left(k_{-2}+k_{+3}\right)\times \text{Open}\left(t\right)$$

$$\text{dOpen}\cdot M\cdot \text{ADP}\cdot P_{i}\left(t\right)/dt=k_{+3}\times \text{Open(t)}+k_{-4}\times \left[P_{i}\right]\left(t\right)\times \text{Open}\cdot M\cdot \text{ADP}-\left(k_{-3}+k_{+4}\right)\times \text{Open}\cdot M\cdot \text{ADP}\cdot P_{i}\left(t\right)$$

$$\text{dOpen}\cdot M\cdot \text{ADP}\left(t\right)/dt=k_{+4}\times \text{Open}\cdot M\cdot \text{ADP}\cdot P_{i}\left(t\right)-\left(k_{-4}\times \left[P_{i}\right]\left(t\right)+k_{+5}\right)\times \text{Open}\cdot M\cdot \text{ADP}\left(t\right)$$

**Table 1:** Constants used in modeled contractions

Parameter	Value	Reference
k_+1_	1.15 × 10^8^ M^−1^ s^−1^	Robertson et al. (1981)
k_−1_	227 s^−1^	McKillop and Geeves (1993)
k_+2_	250 s^−1^	Based on McKillop and Geeves (1993)
k_−2_	1000 s^−1^	Based on McKillop and Geeves (1993)
k_+3_	1050 s^−1^	Based on McKillop and Geeves (1993)
k_−3_	135 s^−1^	Coupland et al. (2001)
k_+4_	1000 s^−1^	Coupland et al. (2001)
k_−4_	60,000 M^−1^ s^−1^	Coupland et al. (2001)
k_+5_	10 s^−1^	Coupland et al. (2001)

**Figure 3:**
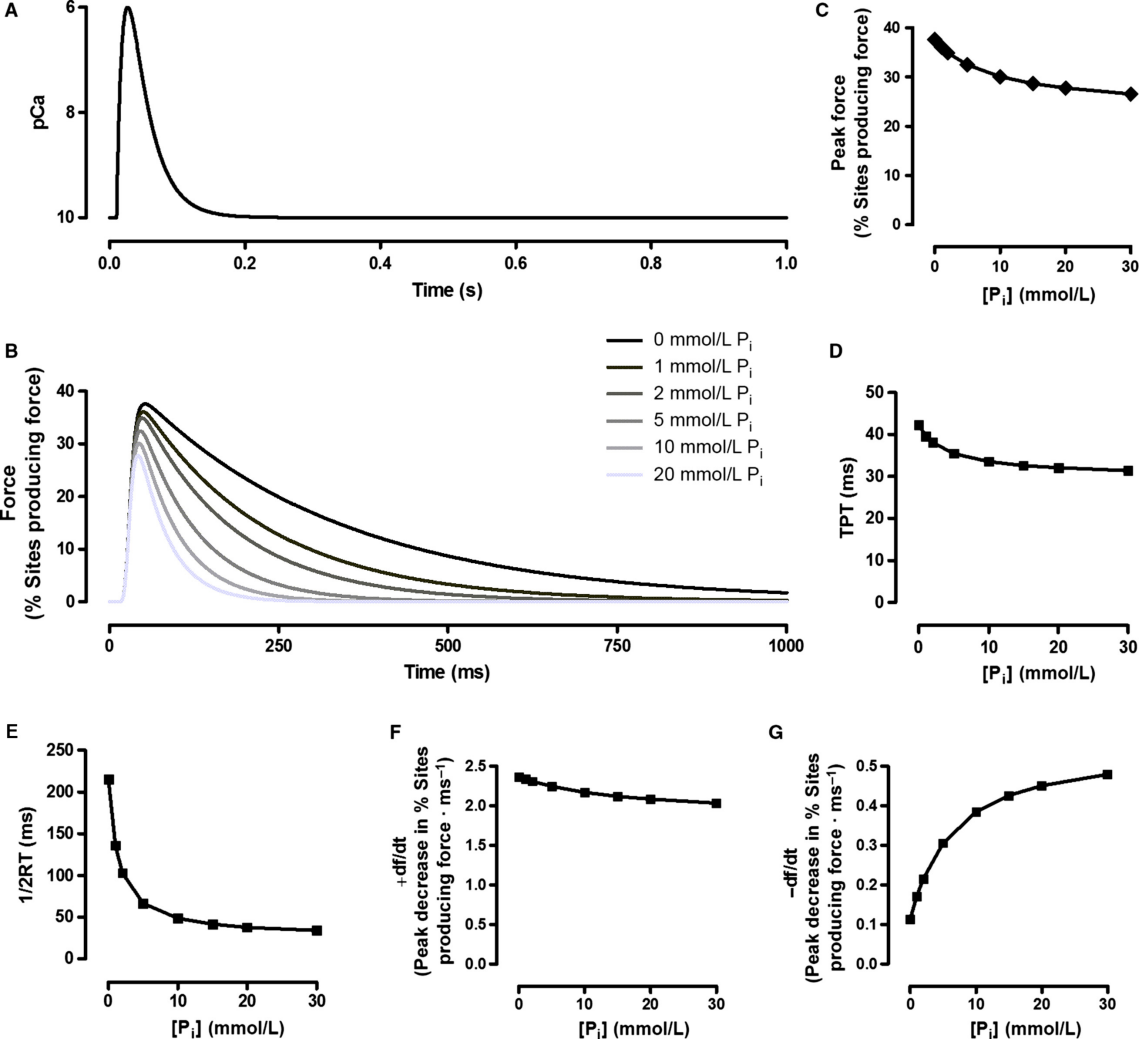
The influence of P_i_ on simulated twitch contractions. All simulated twitches were evoked using the pCa tracing seen in Panel A. Tracings of simulated twitches at different [P_i_] are shown in Panel B. The forces are expressed as the percentage of the total number of crossbridges which are in force‐producing states (Open+Myosin·ADP and Open+Myosin·ADP·P_i_). Panels C–G illustrate the concentration‐dependent influence of P_i_ on peak force (C), time to peak tension (TPT; D), half‐relaxation time (1/2RT; E), peak rate of force production (+d*f*/d*t*; F), and peak rate of relaxation (−d*f*/d*t*; G) of the simulated twitches.

In this model, P_i_ causes force reductions by promoting the reversal of the P_i_ dissociation step (k_−4_), causing accumulation in the Open·M·ADP·P_i_ state, facilitating reversal of the force‐producing step (k_−3_) by mass action (see also Hibberd et al. 1985). P_i_ and Ca^2+^ concentrations were considered time‐dependent values. We approximated the pCa transient during a twitch using the following equation from Robertson et al. (1981):

$$\text{pCa}\left(t\right)={\text{pCa}}_{\mathrm{relaxed}}-A\times \left(e^{-t/f}-e^{-t/r}\right)$$

where pCa = −log_10_[Ca^2+^], pCa_relaxed_ is the steady‐state pCa when relaxed (assumed to be 10), *A* is an amplitude factor (10.39), and *f* (0.01 sec) and *r* (0.03 sec) are the time constants of the fall and rise in pCa, respectively (note that a fall in pCa is a rise in [Ca^2+^]). The resulting pCa curve is shown in Figure 3A. To simulate the fluctuations in pCa during an unfused tetanic contraction, identical twitch pCa curves were initiated at 50 ms intervals (20 Hz), with the rises in [Ca^2+^] above pCa_relaxed_ from overlapping pCa transients considered additive. The resulting pCa tracing is shown in Figure 6A. In simulated contractions where [P_i_] was increased, P_i_ was set to begin rising at the same time as the initial increase in Ca^2+^, increase linearly for 2.0 sec, and then remain constant at the new level for the remainder of the contraction.

To investigate how the duration of the twitch pCa transient might influence the relationship between twitch duration and [P_i_], transients of varying lengths were generated by varying the *r* value from the equation above between 0.011 and 4, and then adjusting the A value to maintain a peak pCa value of 6.00.

Each differential equation shown above is in the form dS_*t*_/d*t* = *f*(S_*t*_,*t*), where S_*t*_ is the population of the crossbridge state at time *t*. As dS_*t*_/d*t* is the instantaneous slope of *f*(S_*t*_,*t*), this equation can be rewritten as (S_*t*+1_ − S_*t*_)/(*t* + 1 − *t*) = *f*(S_*t*_,*t*). The population of crossbridge state S at time *t* + 1 can then be approximated as S_*t*+1_ = S_*t*_ + *f*(S_*t*_, *t*) × (*t* + 1 − *t*) (Euler's method), with initial conditions set such that all crossbridges begin in the blocked state. We used a value of 0.1 ms for the time interval (*t* + 1 − *t*). Using reaction step 1 as an example:

$$\text{[Blocked] at 0.1 ms = [Blocked] at 0.0 ms}+(k_{-1}\times \text{[Open] at 0.0 ms}-k_{+1}\times \left[{\text{Ca}}^{2+}\right]\times \text{[Blocked] at 0.0 ms)}\times (\text{0.1 ms ‐ 0.0 ms)}$$

As everything on the right side of the equation is known from the initial conditions where all sites are blocked, the [Blocked] at 0.1 ms can be estimated by this method, as can the concentration of sites in all other states. After obtaining the estimated populations of all five states at 0.1 ms, these values were used to estimate the populations of all states at 0.2 ms and so forth. Using this method, our simulations were run for 3.0 sec. Flux through each reaction step at each time point was calculated as k_+x_·*n*_i_ − k_‐x_·n_j_ where *n*_i_ and *n*_j_ are the populations of crossbridge states *i* and *j*, and k_+x_ and k_−x_ are the forward and reverse rate constants for reaction step x as shown in Table 1, accounting for [Ca^2+^] or [P_i_] as appropriate for the second‐order reaction steps (Linari et al. 2010).

### Mouse experimental protocols

#### Mouse characteristics

Experiments were performed on soleus and EDL muscles of female C57BL/6 mice (*n* = 14), aged 123 ± 10 days, and weighing 24.6 ± 0.8 g. All testing was done at the University of Waterloo in the laboratory of A.R.T. and all protocols and procedures were approved by the University of Waterloo Committee for Animal Care and are consistent with the guidelines established by the Canadian Council on Animal Care.

#### Muscle preparation and experimental procedures

Following death by cervical dislocation, soleus and EDL muscles were excised with tendons intact and kept on ice in a dissecting solution (containing in mM: 136.5 NaCl, 5.0 KCl, 11.9 NaHCO_3_, 1.8 CaCl_2_, 0.40 NaH_2_PO_4_, 0.10 EDTA, and 0.50 MgCl_2_, pH 7.3). EDL muscles were mounted in a 1200A in vitro test system (Aurora Scientific Inc., Aurora, ON, Canada) between a dual‐mode servomotor (model 300C, Aurora Scientific Inc) and a fixed platform. Soleus muscles were mounted between a F30 type 372 force transducer (Harvard Apparatus, Saint‐Laurent, QC, Canada) and a fixed platform in a TIOX test chamber (Hugo Sachs Elektronik, March, Germany and Harvard Apparatus) which had been modified to allow constant gas perfusion. Both soleus and EDL muscles were incubated for 30 min in the experimental solutions at a length just enough to have some resting tension. Optimum length for force production was then found by stimulating the muscle for 300 ms at 150 Hz at supramaximal voltage and current, and increasing length at 30 sec intervals until active force no longer increased between trials. A 0.2 ms pulse width was used for all stimuli. Bath temperature was held constant at 30°C. The experimental solutions were constantly perfused with a 95% O_2_, 5% CO_2_ gas mixture. Stimulation was computer controlled and applied via flanking platinum electrodes using either a model 701C stimulator (EDL; Aurora Scientific Inc) or a PLUGSYS module (soleus; Hugo Sachs Elektronik). Muscles were then allowed to rest for 30 min before the experiments began. The protocol employed a crossover design where each muscle was stimulated in a pyruvate‐containing solution and a glucose‐containing solution, alternating the order between muscles. The glucose solution contained (in mmol·L^−1^): NaCl, 115; KCl, 5; MgCl_2_, 0.5; CaCl_2_, 2.5; NaH_2_PO_4_, 1; NaHCO_3_, 24; glucose, 11; EDTA 0.5; pH 7 4. The pyruvate solution contained (in mmol·L^−1^) NaCl, 115; KCl, 5; MgCl_2_, 0.5; CaCl_2_, 2.5; NaH_2_PO_4_, 1; NaHCO_3_, 24; sodium pyruvate, 11; EDTA 0.5; pH 7 4. All solutions were oxygenated for 30 min prior to being introduced to the muscle. EDL muscles were stimulated at 5, 10, 15, 20, 25, and 30 Hz, with a 5 min break between frequencies. The soleus muscles were stimulated at 2, 5, 10, 15, 20, and 25 Hz, with a 5 min break between frequencies. A total of 50 pulses were applied at each frequency.

#### Data analysis

The tension developed at each local peak in the unfused tetanic contractions was calculated. The highest of these local peaks was designated as the early tension peak, or “apex.” The apex was limited to the first 15 stimuli to minimize the confounding influence of potentiation by myosin regulatory light chain phosphorylation that was sometimes seen later in the protocols. Sag was defined as the difference in tension between the apex and the lowest tension peak that followed the apex (see Fig. 1). This procedure was applied regardless of whether or not summation was observed during the contraction.

#### Statistics

Data were analyzed using two‐way repeated measures ANOVAs (stimulation number and incubation condition) with Tukey's honest significant difference post hoc test performed where appropriate. Peak changes in relaxation rates and relaxation times were performed using Student's *t*‐test for paired samples. Values are reported as mean ± standard error throughout the manuscript, and differences were considered significant at *α *= 0.05. No statistical analyses were performed on the modeled results.

## Results

### Simulated contractions

**Figure 4:**
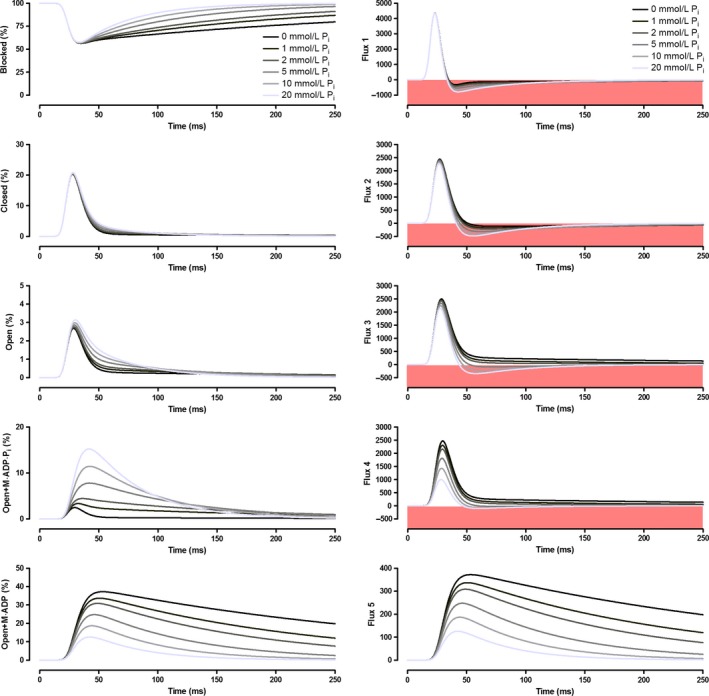
P_i_‐dependent population of each of the crossbridge states (left) and net flux through the reaction steps (right) during the simulated twitch contractions. Negative flux (pink regions) corresponds to a smaller flux through k_+i_ than through k_−i_, where i refers to the reaction steps labeled in Figure 2.

Our model was designed to demonstrate how changing [P_i_] might influence crossbridge cycling and the time‐dependent parameters of contractions which cause force to sag during unfused tetani. The influence of different [P_i_] on our modeled twitch parameters are illustrated in Figure 3. The P_i_‐sensitivity of twitch force, time to peak tension (TPT), half‐relaxation time (1/2RT), peak rate of force production (+d*f*/d*t*), and peak rate of relaxation (−d*f*/d*t*) decreased nonlinearly as [P_i_] increased, with little additional effect seen at [P_i_] greater than 10 mmol·L^−1^. Relaxation parameters were particularly P_i_‐sensitive, with a 323% increase in −d*f*/d*t* and an 84% reduction in 1/2RT between 0 and 30 mmol·L^−1^ P_i_. In contrast, twitch force decreased by 29%, TPT decreased by 25%, and +d*f*/d*t* decreased by 14% between 0 and 30 mmol·L^−1^ P_i_. As P_i_ was increased, the proportion of sites in the Open+M·ADP·P_i_ state increased while the proportion of sites in the Open+M·ADP state decreased (Fig. 4). During twitch relaxation, flux through step 5 of our kinetic scheme decreased as P_i_ was increased, while reaction step 3 was driven backwards at [P_i_] 5 mmol·L^−1^ and above.

**Figure 5:**
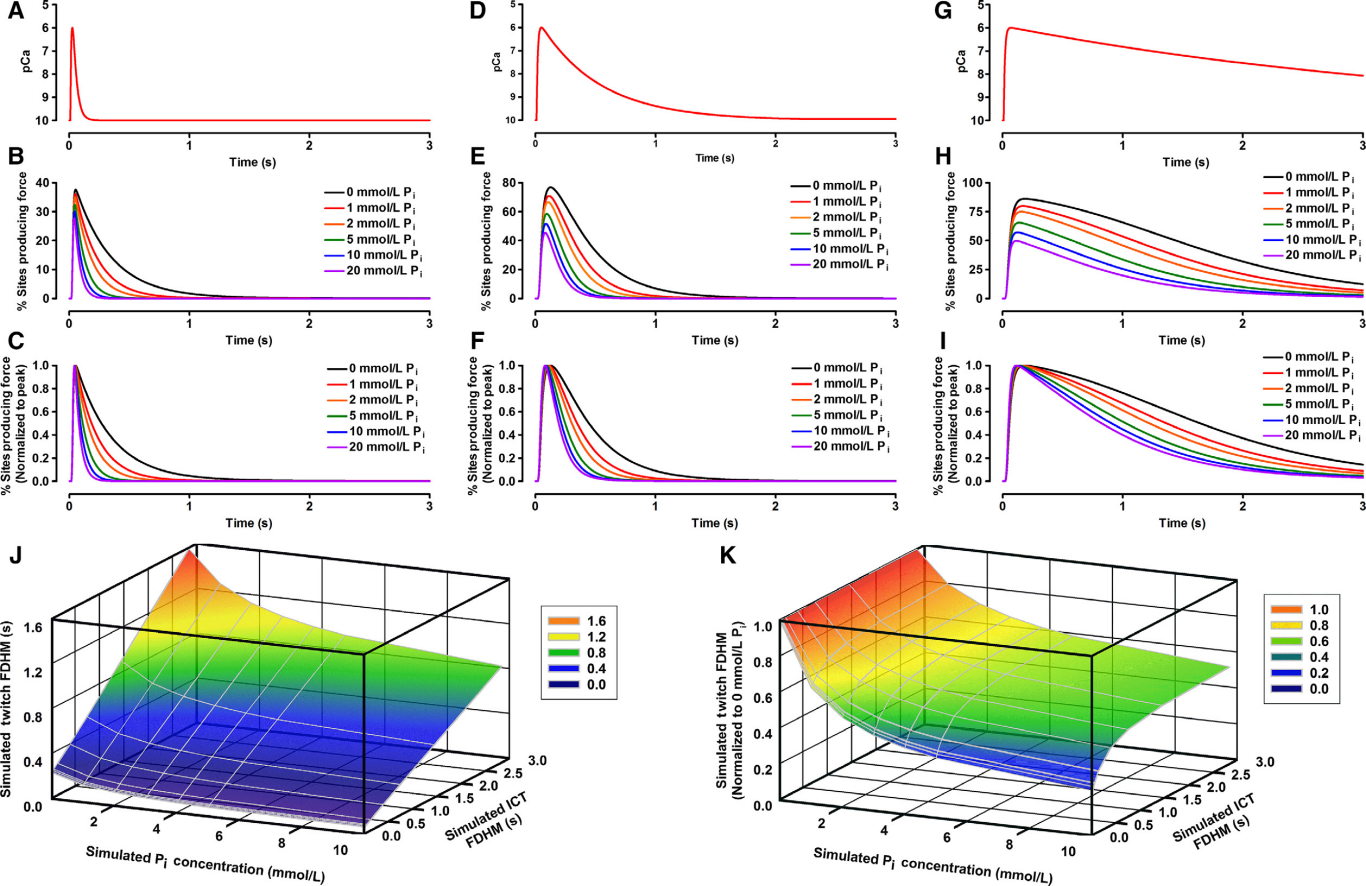
The influence of intracellular Ca^2+^ transient (ICT) duration on the duration of simulated twitches at various [P_i_]. A, D, and G depict simulated intracellular Ca^2+^ transients of varying durations generated as described in the methods. B, E, and H depict the twitches generated at different [P_i_] from the ICTs depicted in A, D, and G, respectively. C, F, and I depict the twitches from B, E, and H, respectively, normalized to the peak proportion of sites producing force in each simulated twitch. Summary data depicting how twitch duration (full duration at half maximum; FDHM) varies with ICT duration (FDHM of ICT in pCa units) and [P_i_] are shown in raw time values (J) and normalized to twitch FDHM at 0 mmol·L^−1^ P_i_ (K).

Twitch duration decreased as P_i_ was increased regardless of the duration of the pCa transients, though the effects of P_i_ were more potent for the shorter pCa transients (Fig. 5). For example, twitches evoked using the briefest of our simulated pCa transients (FDHM 25.5 ms) exhibited decreases in FDHM of 82.2% between 0 and 30 mmol·L^−1^ P_i_. In contrast, twitches evoked using the longest of our simulated pCa transients (FDHM 2.84 sec) exhibited decreases in FDHM of 53.2% as P_i_ was raised from 0 to 30 mmol·L^−1^ P_i_.

**Figure 6:**
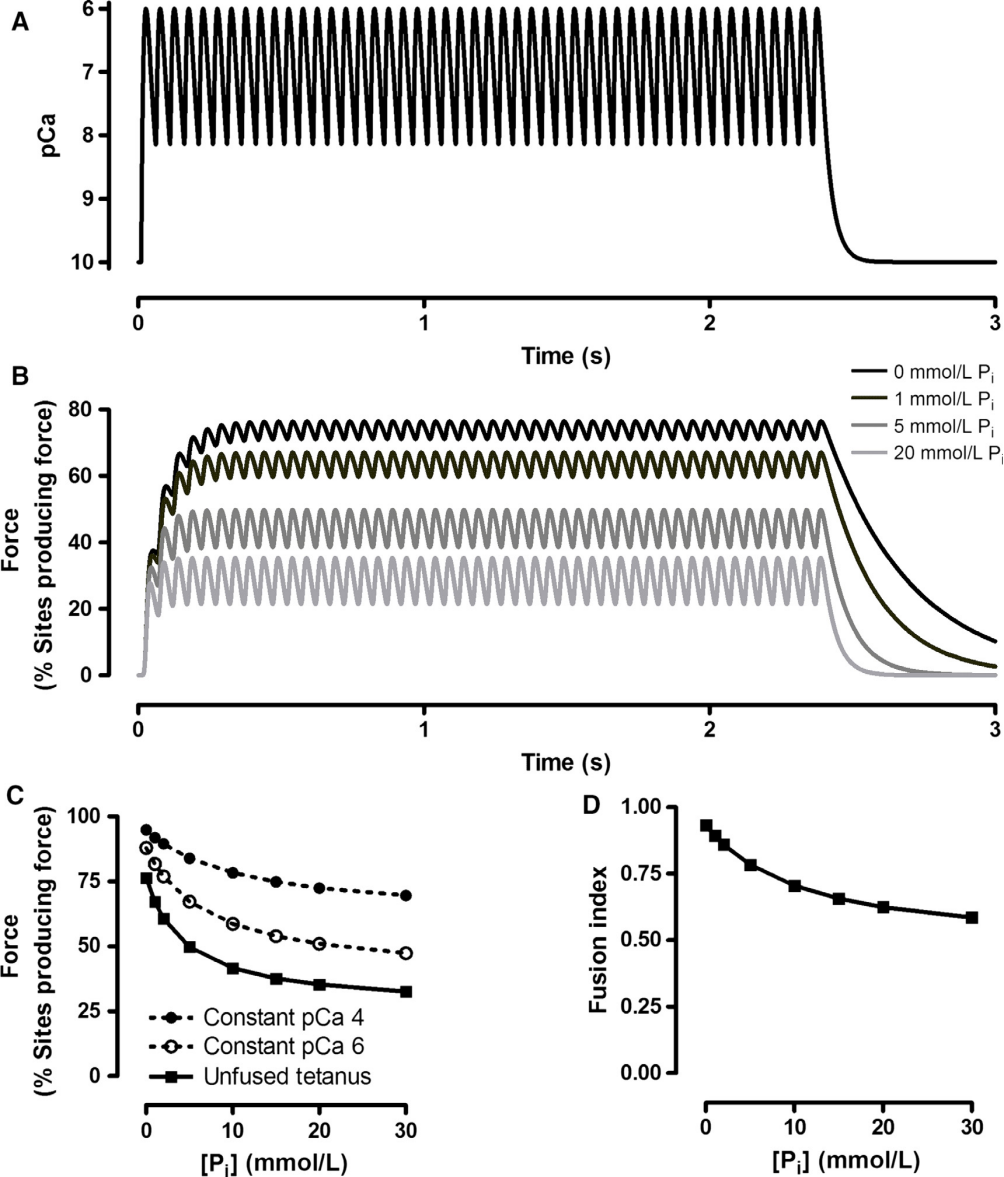
Simulated unfused tetanic contractions with constant P_i_. All contractions were evoked using the Ca^2+^ tracing seen in Panel A. Tracings of the simulated unfused tetani are shown in Panel B. The forces are expressed as the percentage of the total number of crossbridges which are in force‐producing states (Open+Myosin·ADP and Open+Myosin·ADP·P_i_). The relationship between [P_i_] and peak force during the unfused tetani is shown in Panel C, along with simulated force‐P_i_ relationships at constant pCa values of 4.0 and 6.0. The influence of P_i_ on the fusion index of the simulated contractions is shown in Panel D. Fusion index was calculated as the number of bound crossbridges at the lowest point of the relaxation phase prior to the final peak in crossbridge binding, divided by the number of bound crossbridges at the final peak (Celichowski and Grottel 1995).

**Figure 7:**
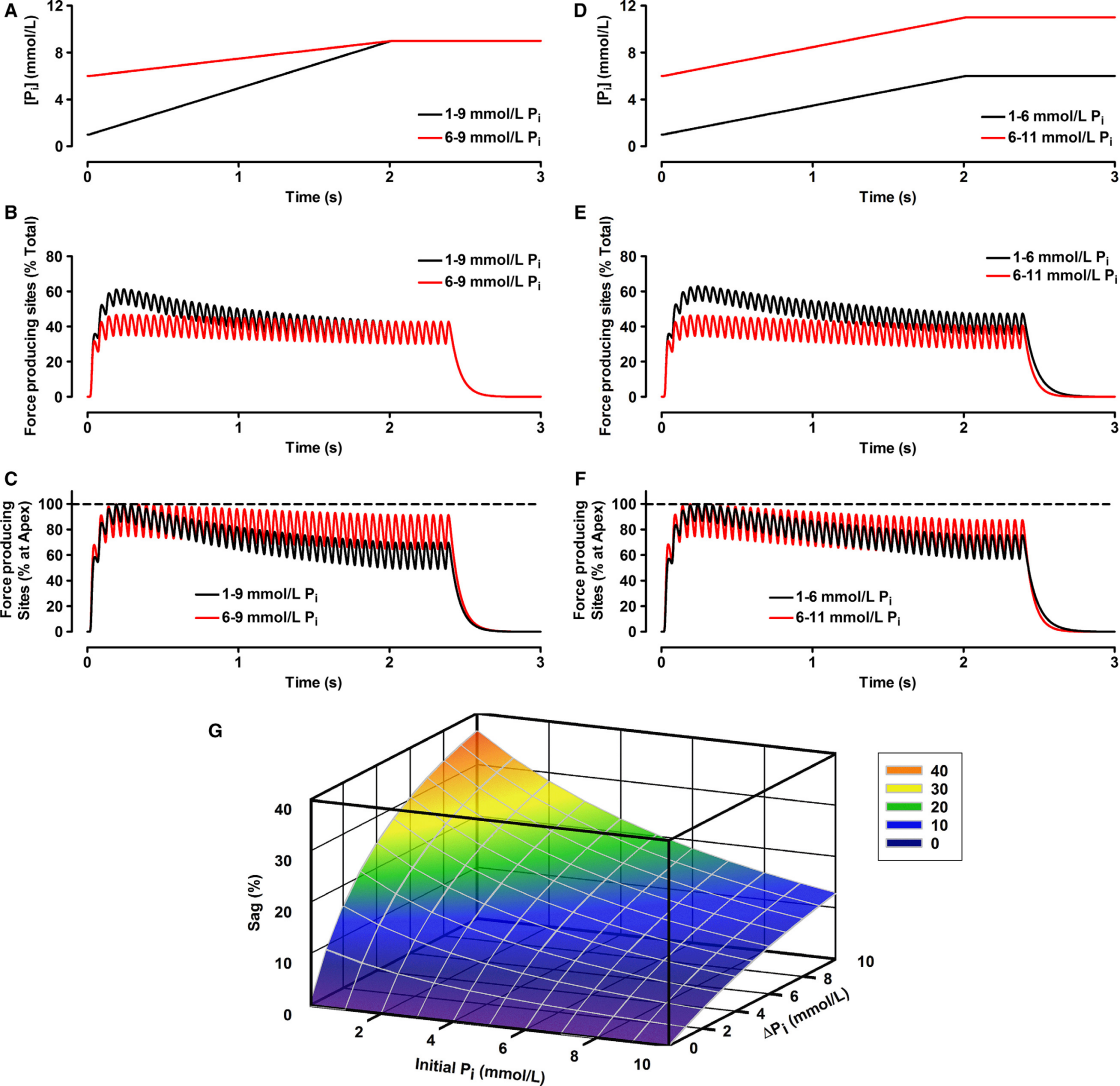
Simulated unfused tetanic contractions with increasing [P_i_]. P_i_ was set to increase linearly at the onset of the Ca^2+^ increase (the Ca^2+^ record is shown in Figure 4A), 0.01 sec into the simulations, and increase for 2.0 sec. Panels A–C demonstrate how sag is increased when initial P_i_ is reduced, but final P_i_ is held constant. Panels D–F demonstrate how sag is reduced when the initial starting concentration is increased, but the magnitude of P_i_ increase is constant. The surface plot in panel G summarizes how sag varied as a function of [P_i_] at the onset of contraction (Initial [P_i_]) and the change in [P_i_] during the contraction (∆[P_i_]).

The P_i_‐mediated decrease in contraction duration (sum of twitch TPT and 1/2RT) resulted in less fusion during simulated unfused tetanic contractions, as measured by the fusion index (Celichowski and Grottel 1995) (Fig. 6). When P_i_ was held constant during the simulated contractions, there was no sag present. To cause sag, it was necessary to increase the [P_i_] during the contractions. The sample tracings shown in Figure 7 illustrate how the magnitude of P_i_ increase and the initial [P_i_] influenced the shape of the contractions. Sag was most sensitive to changes in [P_i_] when the [P_i_] was low initially. These results are summarized in the surface plot seen in Figure 7G. For any given initial and final [P_i_], sag was greater when onset of P_i_ increase was delayed, or if the P_i_ increase was spread over a longer time period; both these factors enhanced summation early in the contraction, resulting in higher force at the apex with no effect on the force during the plateau with constant P_i_ (not shown). Collectively, this model demonstrates how known influences of P_i_ on crossbridge function can influence the kinetics of muscle contraction and cause varying degrees of sag.

### Mouse experiments

#### Soleus contractile parameters

**Figure 8:**
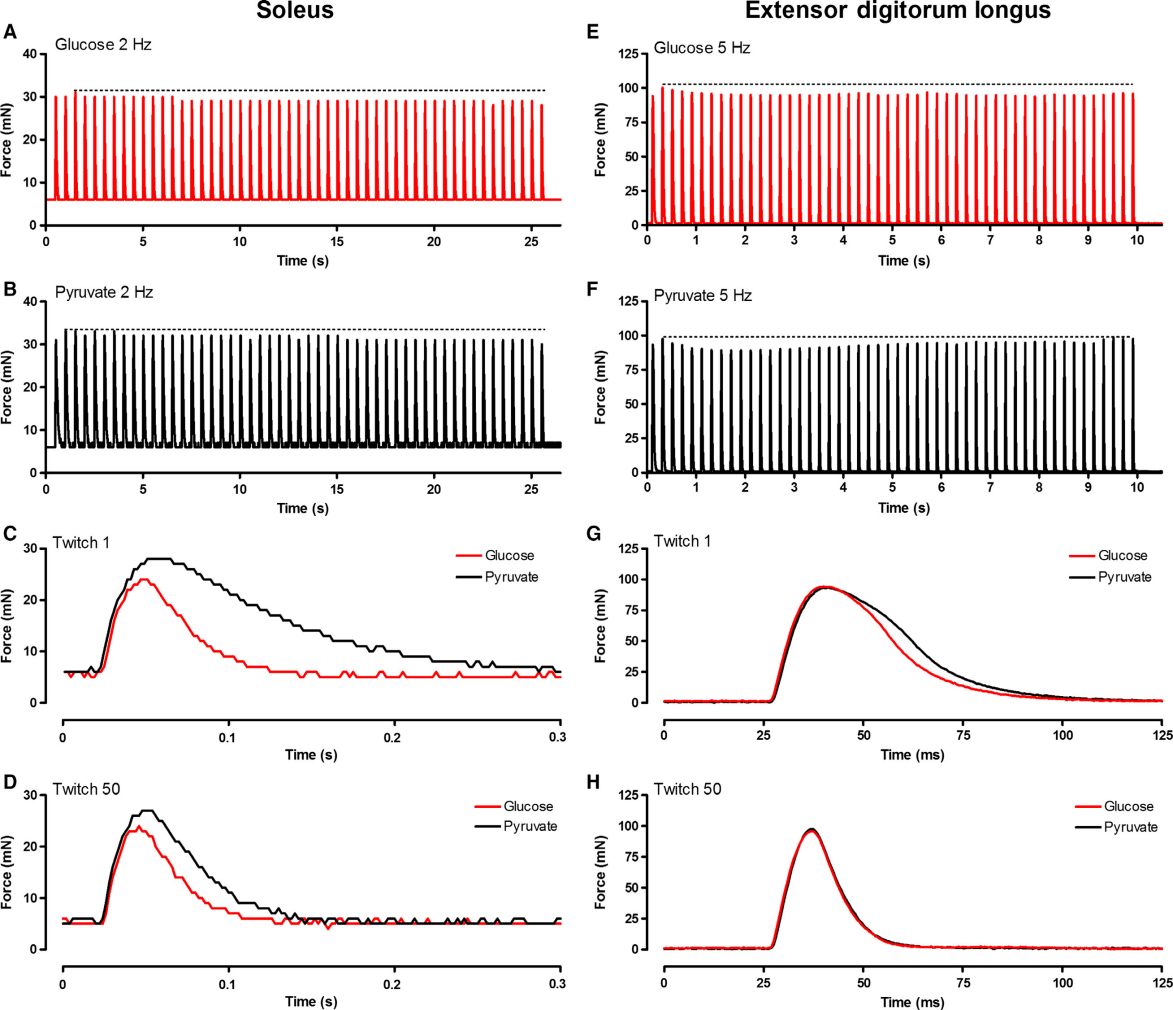
Representative raw force tracings from repeated twitches evoked in soleus and EDL muscles. In panels A, B, E, and F, the dashed horizontal line corresponds to the apex in force achieved during the contractions. The first (C, G) and final (D, H) twitches in the series of contractions depicted in Panels A and B (soleus) and Panels E and F (EDL) are shown in greater detail.

Pyruvate significantly increased (*P *<* *0.05) twitch force, time to peak tension (TPT), half‐relaxation time (1/2RT), and significantly decreased (*P *<* *0.05) the peak rate of relaxation (−d*f*/*dt*) in the soleus muscle relative to the glucose condition (Table 2). Pyruvate did not affect the peak rate of force production (+d*f*/d*t*) in the soleus. Summary data are shown in Table 2, and sample traces are shown in Figure 8A–D.

**Table 2:** The effects of metabolic substrate on twitch parameters

Substrate	Glucose			Pyruvate		
Twitch number	1	50	% Change	1	50	% Change
EDL						
Peak tension (mN)	52.3 ± 5.2	59.6 ± 5.3^a^	16.4 ± 4.5	52.6 ± 6.1	57.2 ± 6.0^a^	11.4 ± 2.8
+d*f*/d*t* (mN ms^−1^)	8.33 ± 0.82	9.98 ± 0.93^a^	21.4 ± 2.6	7.92 ± 0.80	9.41 ± 0.91^a^	19.7 ± 2.6
TPT (ms)	11.3 ± 0.3	9.0 ± 0.1^1^	−19.7 ± 1.9	11.7 ± 0.5(^†^)	9.3 ± 0.1^a^	−19.0 ± 3.1
−d*f*/d*t* (mN ms^−1^)	−2.96 ± 0.23	−5.54 ± 0.43	90.3 ± 9.1	−2.36 ± 0.26^b^, ^a^	−5.16 ± 0.47^b^, ^a^	127.1 ± 9.3^c^
1/2RT (ms)	14.6 ± 0.6	6.9 ± 0.4^a^	−52.6 ± 2.0	19.8 ± 1.0^b^	7.0 ± 0.3^a^	−63.5 ± 2.7^c^
Soleus						
Peak tension (mN)	25.6 ± 2.4	24.3 ± 2.4(*)	−5.0 ± 2.2	31.7 ± 4.8^b^	28.1 ± 3.0^a^^,^ b	−7.1 ± 2.9
+d*f*/d*t* (mN ms^−1^)	1.54 ± 0.14	1.70 ± 0.15^a^	11.0 ± 2.8	1.52 ± 0.17	1.63 ± 0.16^a^	8.2 ± 1.5
TPT (ms)	23.8 ± 1.2	15.5 ± 6.8^a^	−34.5 ± 2.3	33.0 ± 2.8^b^	20.8 ± 1.5^a^^,^ b	−35.2 ± 4.1
−d*f*/d*t* (mN ms^−1^)	0.45 ± 0.03	0.49 ± 0.03^a^	10.3 ± 4.0	0.34 ± −0.03^b^	0.47 ± 0.03^a^	41.9 ± 5.2^c^
1/2RT (ms)	42.5 ± 3.9	34.0 ± 2.0^a^	−16.2 ± 4.5	69.2 ± 7.2^b^	41.7 ± 4.0^a^^,^ b	−37.9 ± 3.2^c^

% Change was calculated as (value at twitch 50 – value at twitch 1) × (value at twitch 1)^−1^.

a For the same substrate, Twitch 1 ≠ Twitch 50; *P *<* *0.05; (*)*P *<* *0.1.

b For the same Twitch Number, Glucose ≠ Pyruvate; *P *<* *0.05; (†) *P *<* *0.1.

c % Change in Glucose ≠ % Change in Pyruvate; *P *<* *0.05.

Regardless of incubation condition, 50 twitch contractions applied at 2 Hz resulted in lower (*P *<* *0.05) peak tension, TPT, and 1/2RT relative to initial values, while +d*f*/d*t* and −d*f*/d*t* were increased (*P *<* *0.05) relative to initial values. The relative changes in −d*f*/d*t* were fourfold greater in pyruvate than in glucose, and 2.3‐fold greater changes were seen in 1/RT when soleus was incubated in pyruvate than in glucose. The contraction‐induced changes in peak tension, TPT, and +d*f*/d*t* were similar between the glucose and pyruvate conditions.

**Figure 9:**
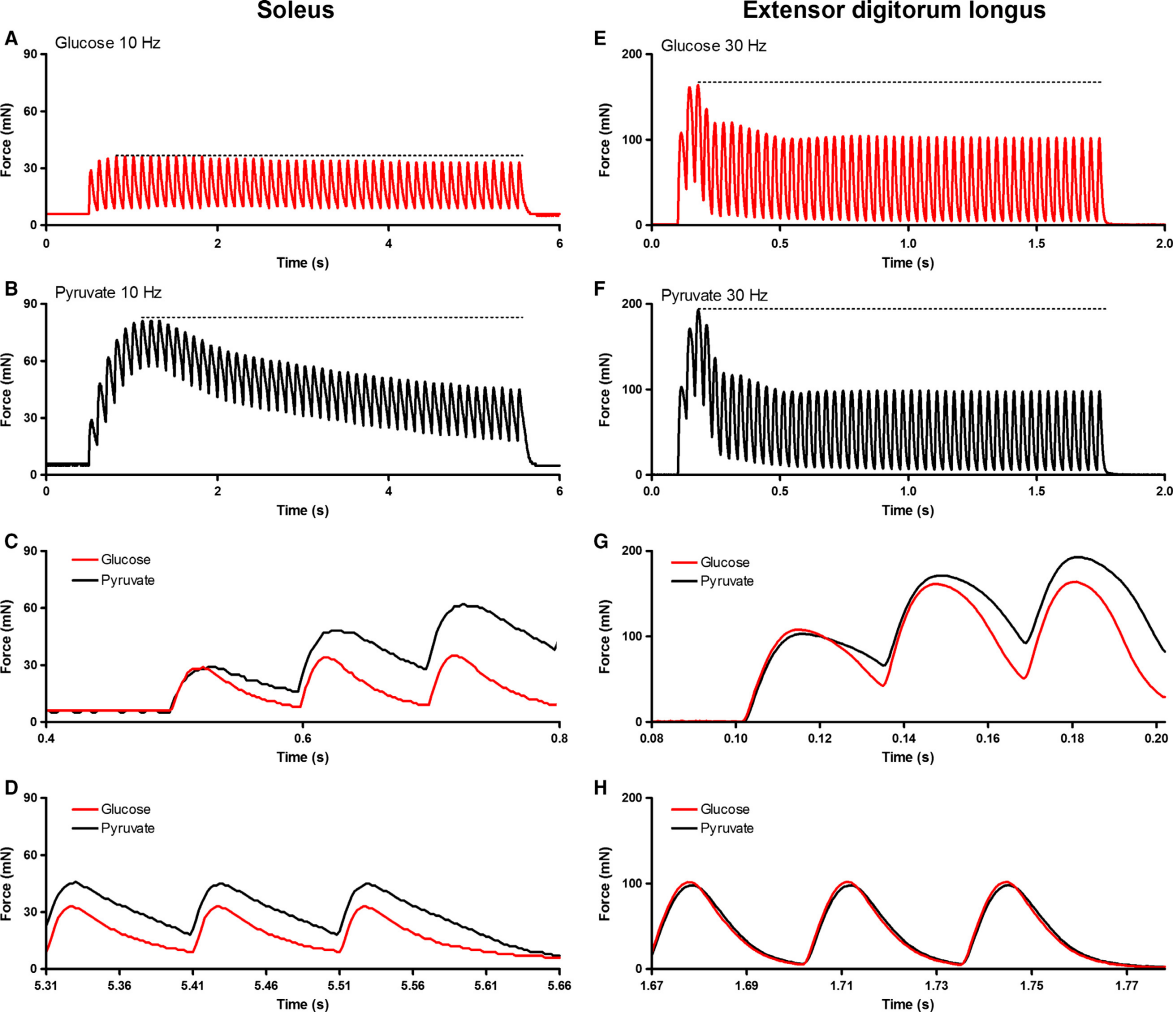
Representative raw force tracings from unfused tetani evoked in soleus and EDL muscles. In panels A, B, E, and F, the dashed horizontal line corresponds to the apex in force achieved during the contractions. The first three (C, G) and last three (D, H) stimuli of each contraction depicted in Panels A and B (soleus) and Panels E and F (EDL) are shown in greater detail.

**Figure 10:**
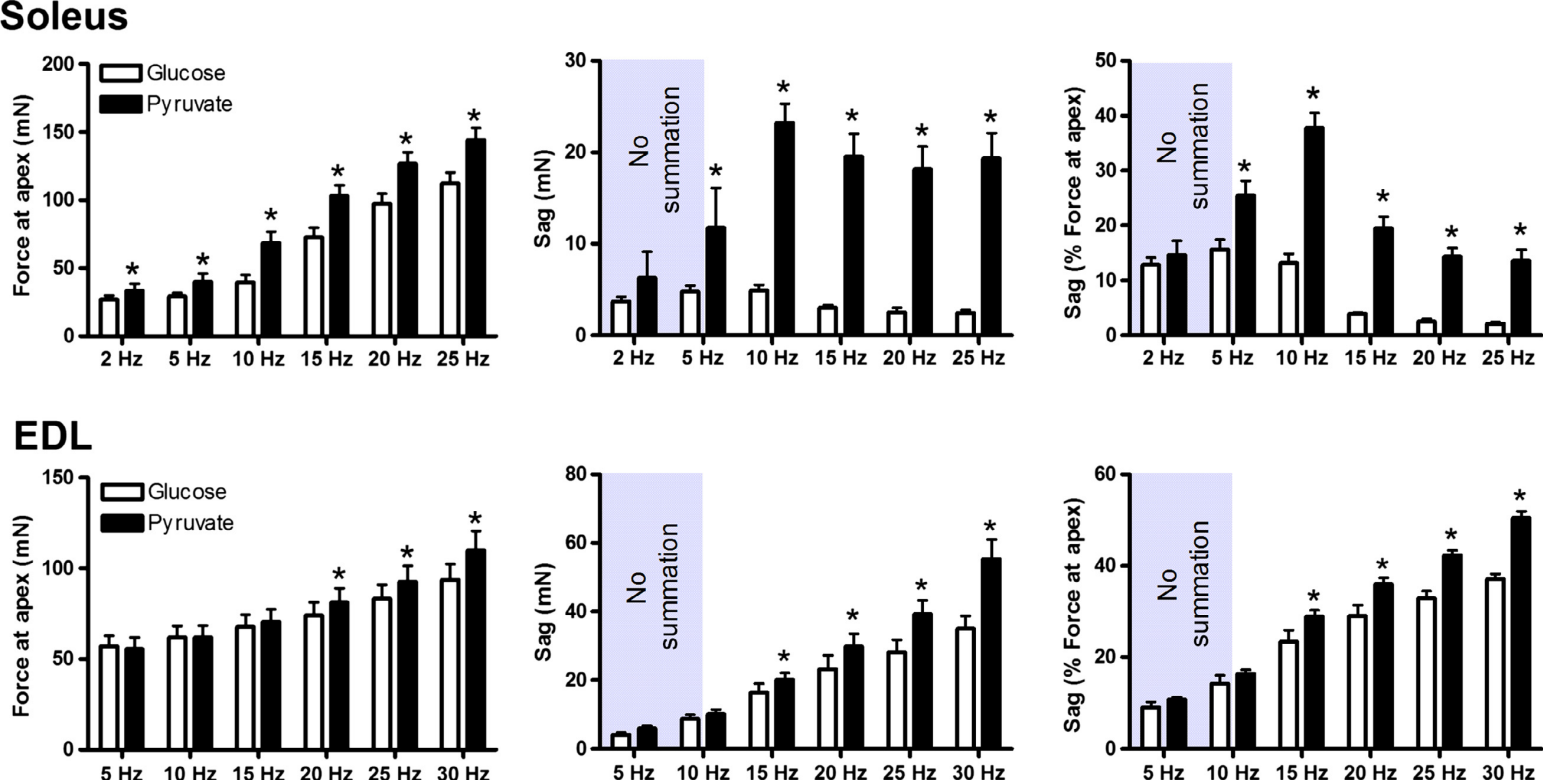
The influence of pyruvate on force at the apex (left), sag in terms of absolute force (middle) and sag relative to the force at the apex (right) for the soleus (top) and EDL (bottom). *Value is significantly different from the glucose condition at the same frequency (*P *<* *0.05). Values are mean ± SEM.

In glucose, soleus was resistant to sag (Figs. 9 and 10). In glucose, the stimulation frequencies eliciting the most sag (12–16%) were 2, 5, and 10 Hz where there was little to no summation, indicating that this sag was predominantly due to declines in twitch force and not declines in summation. At 15, 20, and 25 Hz where summation was more prominent, contractions exhibited sag of only 2–4%. In pyruvate, however, sag was a prominent feature of the contractions at stimulation frequencies 5 Hz and above, peaking at 38 ± 3% at 10 Hz. The increased sag in pyruvate resulted from improved summation early in the contractions, resulting in higher (*P *<* *0.05) force at the apex in pyruvate than glucose at each of the frequencies tested. The sag values we report for pyruvate underreport the true values as force was still converging toward a lower plateau after 50 contractions in each of the stimulation frequencies.

#### EDL contractile parameters

Relative to glucose incubation, pyruvate incubation significantly (*P *<* *0.05) decreased −d*f*/d*t* and significantly (*P *<* *0.05) increased 1/2RT relative to glucose incubation. TPT also tended to be higher in pyruvate than glucose, but this failed to reach significance (*P *=* *0.07). Peak tension and +d*f*/d*t* were not affected by the different incubation conditions. Summary data are shown in Table 2, and sample traces are shown in Figure 8E–H.

After 50 twitches evoked at 5 Hz, twitch force, +d*f*/d*t*, and −d*f*/d*t* were increased (*P *<* *0.05), while 1/2RT and TPT were reduced (*P *<* *0.05) relative to initial conditions. The magnitude of change in peak tension, +d*f*/d*t*, and TPT were similar (*P *<* *0.05) between pyruvate and glucose conditions. Both relaxation parameters exhibited greater changes in pyruvate than glucose, with proportional changes of 40% and 21% larger in −d*f*/d*t* and 1/2RT, respectively.

The EDL was prone to sag in both glucose and pyruvate (Figs. 9 and 10). Both absolute and relative values of sag increased as stimulation frequency was increased from 5 to 30 Hz. Sag was significantly (*P *<* *0.05) higher in pyruvate than glucose at frequencies of 15, 20, 25, and 30 Hz. Force at the apex of the contractions was greater (*P *<* *0.05) in pyruvate than glucose at 20, 25, and 30 Hz, but not at 5, 10, or 15 Hz.

## Discussion

The shape of the unfused tetanus is determined by a highly complex set of interacting factors which compete to influence force through their effects on crossbridge binding and dissociation. The phenomenon of sag is influenced primarily by a contraction‐induced reduction in the duration of the contraction, an effect which until very recently has been suspected to be caused by accelerated cytosolic Ca^2+^ removal. Our previous work demonstrating that contraction time decreases during repetitive stimulation, without concomitant reductions in the duration of the intracellular Ca^2+^ transient (Smith et al. 2013b, 2014) projected considerable doubt on this theory. In this study, we provide a theoretical framework and preliminary experimental evidence which suggest that sag can be explained by differences in [P_i_]_c_ prior to contraction and changes in [P_i_] during contraction. Though we did not measure P_i_ directly, our theoretical and experimental data fit a scenario in which increased [P_i_]_c_ is associated with reductions in twitch tension, TPT, 1/2RT, and the peak rate of force production. In contrast, the peak rate of relaxation is enhanced by P_i_, leading to an abbreviation of contraction duration which reduces the level of fusion during unfused tetanic contractions at any given frequency. In both the modeled and experimental results, P_i_ most robustly influenced relaxation parameters. Collectively, our results support a mechanism where a contraction‐induced increase in [Pi] reduces the average time crossbridges spend in force producing states following binding. This change in crossbridge kinetics reduces contraction duration and impairs fusion later in the contraction, resulting in sag. This study provides the first experimental evidence of a fast‐acting, intrinsic cellular mechanism capable of rapidly decreasing contraction duration during the course of an unfused tetanus. The specificity of the P_i_ effect can be localized to the crossbridge since P_i_ does not directly influence the Ca^2+^‐affinity of troponin C (TnC) (Palmer and Kentish 1994), and P_i_ slows the activity of the Ca^2+^ pump (Allen and Westerblad 2001), an effect which would oppose sag. This proposed mechanism is particularly appealing for its simplicity, as the myosin ATPase nucleotide‐binding domain represents both a source and the active site of P_i_, thereby guaranteeing a localized myofibrillar response during a contraction.

At stimulation frequencies where twitch contractions did not overlap and summate, the sag seen was exclusively due to a decline in twitch force. At higher stimulation frequencies where twitches summated, both declining twitch force and shortening contraction durations contributed to the sag response. The declines in twitch force are presumably due to a combination of P_i_‐mediated declines in the number of force‐producing crossbridges, though contraction‐induced declines in the amplitude of the intracellular Ca^2+^ transient may have also played a role (Smith et al. 2014).

One of the most intriguing features of sag is its fiber‐type dependence, where sag is often present in fast muscle but small or absent in slow muscle (Burke et al. 1973; Burke 1990; Grottel and Celichowski 1990; Bigland‐Ritchie et al. 1998; Carp et al. 1999; Celichowski et al. 1999, 2005; Krutki et al. 2006). Our data suggest that the resting [P_i_] is a critically important factor in determining whether sag is present or absent makes it possible to explain at least some of the fiber‐type‐dependent properties of sag, as the resting [P_i_] of fast muscle is lower than that of slow muscle (Kushmerick et al. 1992; Phillips et al. 1993; Dahlstedt et al. 2000). It is well documented that tension becomes less sensitive to changes in P_i_ as the [P_i_] increases (Pate et al. 1998). Our modeled data indicate that all relevant twitch parameters also become less sensitive to changes in [P_i_] as [P_i_] was increased from 0 to 30 mmol·L^−1^. This finding leads to the hypothesis that under conditions with a high initial [P_i_], contraction duration may not decrease enough during an unfused tetanus to cause a large sag response, even with a substantial increase in [P_i_]. Assuming that resting [P_i_]_c_ was lower in our pyruvate condition than in our glucose condition (Phillips et al. 1993; Mallet and Sun 1999), we can surmise that lowering [P_i_] prior to stimulation allows greater reductions in contraction duration and more sag during unfused tetani.

Sag is not considered well suited to differentiate between fast and slow motor units of human muscle, as sag is frequently absent from human muscle (Macefield et al. 1996; Bigland‐Ritchie et al. 1998). However, there are some clear examples of sag in electrically evoked contractions in human muscle (e.g., Booth et al. 1997; Vøllestad et al. 1997; Fowles and Green 2003) which indicates that this phenomenon is relevant to the dynamics of human muscle contraction. Why humans differ from animals in their display of sag may be related to the high levels of resting phosphate reported for human muscle which appears relatively homogeneous between muscles of differing fiber types. For example, an investigation comparing [P_i_] in human adductor pollicis and first dorsal interosseous muscles revealed high [P_i_] (>8 mmol·L^−1^) in both muscles, and no difference between muscles (Turner et al. 1992), despite there being differences in fiber‐type distribution (adductor pollicis 80% type 1 versus first dorsal interosseous 57% type 1) (Johnson et al. 1973). It has also been shown that cats have higher P_i_:PCr and P_i_:ATP ratios in muscles with higher proportions of type II fibers, but this does not occur in human muscles (Meyer et al. 1985; Vandeborne et al. 1993; Takahashi et al. 1996).

Our simulations suggest that P_i_ loses some of its potency in reducing twitch duration as the duration of the pCa transient increases. Fast‐twitch rat muscle has 6–8 times more Ca^2+^ pumps than human muscle (Everts et al. 1989), and parvalbumin does not aid relaxation in human muscle as it does in small rodents (Heizmann 1984), collectively resulting in a slower rate of cytosolic Ca^2+^ removal in humans. This may blunt any P_i_‐mediated decrease in contraction time and help minimize sag in human muscle. Similarly, a slow rate of Ca^2+^ uptake may prevent sag in slow‐twitch muscle in general as sarco‐endoplasmic reticulum Ca^2+^‐ATPase (SERCA) expression in fast‐twitch muscle is 3–8 times greater than that of slow muscle (Everts et al. 1989; Wu and Lytton 1993; Vangheluwe et al. 2005; Murphy et al. 2009; Smith et al. 2013a). It should be noted that [P_i_] was held constant during our simulations. In an intact physiological system, P_i_ and other metabolic byproducts would rise in concentration as contraction duration increases, and the shape of the pCa transient would change as cytosolic Ca^2+^ buffers become saturated, creating potentially confounding influences for experiments aiming to probe this relationship.

Differences in energy utilization may also contribute to differences in the amount of sag between muscles of different species and fiber types. Since a muscle with a long twitch contraction duration starts to summate at lower stimulation frequencies than a muscle with short twitch duration, the energetic cost of an unfused contraction would presumably differ between these muscles. This idea is supported by the finding that P_i_ increases as a function of stimulation frequency (Kushmerick and Meyer 1985). Therefore, low energy demands associated with the low stimulation frequencies needed to evoke an unfused tetanus in slow muscle may also temper P_i_ accumulation and sag relative to a comparable contraction in a faster muscle.

As we did not measure P_i_ levels, our uncertainty regarding the magnitude of change in [P_i_] during contractions in this study is a limitation. However, ^31^P NMR studies suggest that the elevations in P_i_ are rapid and robust, with Challiss et al. (1989) reporting that [P_i_] increases from approximately 3 *μ*mol·g^−1^ at rest to 21 *μ*mol·g^−1^ during a 3 sec 100 Hz tetanus in rat ankle flexors. Kushmerick and Meyer (1985) report increases in [P_i_] during 1.8 min of low‐frequency stimulation of rat lower limb, with P_i_ increasing from near zero values at rest, up to 12, 16, and 22 *μ*mol·g^−1^ during 2, 4, and 10 Hz stimulation, respectively. Our results showing markedly slower contractions in pyruvate than in glucose are consistent with previous reports in both skeletal (Phillips et al. 1993; Sopariwala et al. 2015) and cardiac (Torres et al. 2013) muscle, and the P_i_‐reducing effects of pyruvate incubations have been confirmed previously (Phillips et al. 1993; Mallet and Sun 1999). However, since we saw increasing kinetic rates with repeated contractions in both soleus and EDL, regardless of incubation condition, it is likely that P_i_ accumulated in all groups. Since the force and kinetics of the contractions in glucose and pyruvate converged toward similar forces and kinetic rates by the end of the 50 pulses, it can be speculated that the contraction‐induced level of P_i_ might be similar between the glucose and pyruvate conditions. This would imply that the relative change in [P_i_] caused by contraction would be higher in the pyruvate condition than in the glucose condition. If this is correct, it would also fit with the prediction of our model that sag increases as the magnitude of change in [P_i_] increased, particularly since this increase would be caused primarily by a lower initial P_i_ level.

There is some uncertainty regarding the effects of pyruvate on the Ca^2+^ signal in skeletal muscle. In cardiac muscle, pyruvate incubation has a potent inotropic effect largely due to its agonistic effects on SERCA activity. This leads to greater Ca^2+^ load in the cardiac sarcoplasmic reticulum, enhanced Ca^2+^ release on activation, and twitch force enhancement by ~50% (Martin et al. 1998; Hasenfuss et al. 2002; Mallet et al. 2005; Torres et al. 2013). Several of our observations suggest that pyruvate does not have similar effects on Ca^2+^ release in skeletal muscle. First, if Ca^2+^ release was increased by pyruvate, we would expect to see an increased rate of force production in both muscles; this did not occur in our experiments. We would also expect to see an increase in twitch force if the Ca^2+^ transient was to be significantly enhanced by pyruvate. We saw no change in twitch force in the EDL and although the soleus twitch force increased by 23% in pyruvate, it took 39% more time to reach peak, suggesting prolongation of activation. Finally, the effects of pyruvate on Ca^2+^ release are primarily mediated via enhancement of SERCA activity, which should accelerate cytosolic Ca^2+^ removal and increase the rate of relaxation in muscles incubated in pyruvate. Our experimental results show the opposite effect, as relaxation was slowed after incubation in pyruvate. Therefore, although pyruvate could have had an effect on the Ca^2+^ transient, we do not believe that it is likely to be a confounding factor in our study.

Although sag is not commonly investigated in mouse muscle, our findings regarding the fiber‐type dependence of sag are consistent with those reported for different motor units in cat and rat muscle (Burke et al. 1973; Burke 1990; Grottel and Celichowski 1990; Bigland‐Ritchie et al. 1998; Carp et al. 1999; Celichowski et al. 1999, 2005; Krutki et al. 2006), as well as the work of González and Delbono (2001) who report sag in the majority of mouse EDL fibers, but no sag in the majority of mouse soleus fibers.

## Conclusions

Our data support a P_i_‐based mechanism which is likely to be the primary cause of sag during unfused tetanic contractions in mouse soleus and EDL. We propose that P_i_ accumulation during the contraction decreases the average binding lifetime of force‐producing crossbridges, thereby reducing contraction duration, impairing summation, and causing force to sag during unfused tetani. This mechanism for sag appears to be critically dependent on two nonlinear relationships. One between sag and the [P_i_] at the onset of the contraction protocol; the other between sag and the magnitude of P_i_ increase during the course of the contraction protocol. These relationships provide a tenable explanation regarding the absence or near absence of sag in slow‐twitch muscles, such that the normally high resting [P_i_]_c_ in slow‐twitch muscles limit the capacity for further P_i_‐based reductions in contraction duration, which greatly diminishes capacity of these muscles to exhibit sag. To confirm or disprove the mechanism we have proposed, careful measurement of muscle P_i_ levels will need to be performed before, during, and after unfused tetanic contractions.

## Conflict of Interest

The authors have no conflicting interests, financial or otherwise, regarding the data presented in this manuscript.
